# A Few Thoughts Respecting Pivot Teeth

**Published:** 1849-10

**Authors:** A. Berry


### A FEW THOUGHTS RESPECTING PIVOT TEETH.

BY A. BERRY, D. D. S.

Dr. E. Taylor's method of cutting off teeth for pivoting with very fine saws, is, in my opinion, a great improvement in inserting pivot teeth. It causes less liability to irritation of the periostea of the sockets and fangs, and requires much less filing to fit the artificial crowns, than the common mode of performing this part of the operation.

Gold wire should always be inserted in the wood for pivots, except in temporary cases. It retains the teeth in place much better than the hickory alone, preventing the pivot from bending in the least after being worn for many years. It is best about the size commonly used to fasten large breast pins, and of sixteen carat gold. The hickory should be tough. That grown in a hilly or mountainous region is preferable; and it is an advantage to have it with the annual growths, thick enough to make a pivot without any of the porous portions between them. It is well to saw off an end of the piece for pivots, and make small holes for the wire with a fine bow-drill, inserting in each a piece of wire only long enough for one or two pivots. Then saw off, split, trim to a proper size, and compress them by drawing through a plate in the usual manner.

Pivot teeth, such as were sometimes manufactured ten years ago, with a fine screw thread cut in their pivot holes instead of having them smooth as usual, are a great desideratum; as such teeth never come off the pivot if it is properly inserted.

